# 

**Published:** 1887-08

**Authors:** 


					﻿Vol VII.
AUGUST, 1887.
NO. 8.
THE
pOMCEOPATHlC pHY^lClAJI,
A MONTHLY JOURNAL OF MEDICAL SCIENCE.
“ He is a freeman whom truth makes free, and all are slaves beside."
“ Seek the Truth: come whence it may, cost what it will.”
EDITED BY
Edmund J. lee, m. d.,
AND
WALTER XI. JANIES, M. D.
PHILADELPHIA:
No. 1123 SPRUCE STREET.
3ST 33 W 3270 3t XZ,
A. L. CHATTERTON & COMPANY, PUBLISHER’S AGENTS.
T»O ISTDO IT t
ALFRED HEATH & CO., Sole Agents for Great Britain,
114 Ebury Street, Eaton Square.
Yearly Subscription, $2.50, invariably in advance. Single numbers, 25 cts.
Entered at the Philadelphia Poet-Office as Second-Class Mail Matter.
				

